# Policy analysis in the field of rare diseases in China: a combined study of content analysis and Bibliometrics analysis

**DOI:** 10.3389/fmed.2023.1180550

**Published:** 2023-05-05

**Authors:** Xiaotao Li, Lan Wu, Lina Yu, Youqin He, Min Wang, Yan Mu

**Affiliations:** ^1^Library, Nanjing University of Aeronautics and Astronautics, Nanjing, China; ^2^Medical Record Department, Eye Hospital of Shandong First Medical University (Shandong Eye Hospital), Jinan, China; ^3^State Key Laboratory Cultivation Base, Shandong Provincial Key Laboratory of Ophthalmology, Shandong Eye Institute, Shandong First Medical University & Shandong Academy of Medical Sciences, Jinan, China; ^4^School of Ophthalmology, Shandong First Medical University, Jinan, China; ^5^Bidding and Procurement Office, Tai’an 88 Hospital, Tai'an, China; ^6^School of Healthcare Security, Shandong First Medical University & Shandong Academy of Medical Science, Jinan, China; ^7^Medical Information Center, Shandong Institute of Medicine and Health Information, Jinan, China

**Keywords:** rare disease, China, policy analysis, policy tools, policy themes, bibliometric analysis

## Abstract

**Introduction:**

The Chinese government has made significant strides in addressing the needs of individuals affected by rare diseases in recent years. This paper aims to provide a comprehensive analysis of national rare disease policies in China from 2009 to 2022, using a mixed-methods approach.

**Methods:**

A two-dimensional analytical framework, which includes policy tools and policy themes, is introduced to analyze the rare disease policies comprehensively. Drawing on the policy tools theory proposed by Rothwell and Zegveld, this paper evaluates the tools used in rare disease policies. Co-word analyses and network analyses are employed to identify key themes in rare disease policies and collaboration among government departments.

**Results:**

The rare disease policy landscape in China is undergoing rapid growth, with an increasing number of government departments involved in policy formulation. However, further collaboration between departments is needed to strengthen these policies. Environment-based and supply-based tools are preferred in rare disease policies. The policy themes can be grouped into four categories: (1) Registration, Approval and Supply of Rare Disease Drugs, (2) Construction of Diagnosis and Treatment System for Rare Diseases, (3) Development and Genericization of Rare Disease Drugs, and (4) Social Security for Patients with Rare Diseases.

**Discussion:**

The study provides valuable insights into the current state of rare disease policies in China and offers suggestions for policy improvement. The results show that the Chinese government has made efforts to address the needs of individuals affected by rare diseases, but there is still room for improvement. The collaboration between government departments needs to be strengthened to achieve better rare disease policies. The findings of this study have implications for other countries with similar healthcare systems and can contribute to a better understanding of the impact of rare disease policies on public health.

## 1. Introduction

Rare diseases, a term encompassing a group of illnesses with low prevalence in the population compared to common diseases, are estimated to affect 263–446 million people worldwide, with 5,000–8,000 known rare diseases (1, 2). Although individual rare diseases have a low prevalence, they often have life-threatening or chronic consequences, resulting in a heavy burden on patients and their families (3, 4). Recognizing the global public health significance of rare diseases, growing calls for action are being made in both high and low/middle-income countries (3, 5–11). In line with China’s Healthy China 2030 strategy, rare diseases have received increased attention in recent years, with an estimated 20 million individuals affected in the country (12). The difficulties in diagnosing, treating, and accessing medications for patients with rare diseases have highlighted the need for improved policy measures, particularly in the area of drug policies (13). A recent study indicated that the accessibility of orphan drugs in China improved from 2017 to 2020, demonstrating the effectiveness of the current policy measures (14). This paper aims to gain a deeper understanding of rare disease policies in China, exploring questions such as: what policy tools are included in rare disease policies, what themes are emphasized, what challenges exist in the field of rare diseases in China, and how policy tools can be selected and combined to improve equal access to healthcare for rare diseases. By answering these questions, this study aims to shed light on the characteristics and logic of rare disease policies in China, contributing to the development of rare disease policy both in China and other countries by enriching research methodology and content, providing references for future policy-making, and offering new perspectives.

Numerous studies have explored the policies surrounding rare diseases in various regions and countries. A systematic literature review employed content analysis to analyze the regulations and policies regarding access to orphan drugs in 35 countries, and identified six major categories of regulation and policy tools: national orphan drug policies, orphan drug designation, marketing authorization, incentives, marketing exclusivity, and pricing and reimbursement (15). Another study assessed the rare disease policies of 11 countries with regards to the key needs of patients in five areas: improving coordination of care, diagnostic resources, access to treatments, patient awareness and support, and promoting innovative research (16). In a literature review, publicly available legislative and rare disease health policy data from 12 Eurasian countries were analyzed in five focus areas: rare disease definition, newborn screening, registries, national plans, access to/reimbursement of orphan medicinal products (17). The existing research on rare disease policy in China focuses on the current state of policy discussion. For instance, a 2012 study explored the incentive policies, medical insurance policies, and social supportive activities for rare diseases and orphan drugs in China (18). In a 2019 study, it was demonstrated that a number of policies have been implemented to improve the accessibility of rare disease drugs in China (13). Three crucial aspects of recent national policies regarding rare diseases in China were described in another study in 2021, namely promoting the improvement of rare disease diagnosis and treatment capabilities nationwide, encouraging the research, development, and production of drugs to treat diseases, improving access to medications for patients with rare diseases (19).

This study aims to provide a comprehensive and systematic examination of rare disease policies in China, taking into account both qualitative and quantitative perspectives. Previous research on rare disease policy has been limited to qualitative analysis, with most studies merely providing an overview of policy text. However, recent advancements in policy analysis research call for a shift toward the application of quantitative methods (20). Our study employs both content analysis and bibliometric analysis to extract policy tools and themes from policy documents and to provide an objective and quantitative insight into rare disease policies in China. Our aim is to contribute to the development of policy science and to offer a new paradigm for public policy analysis.

## 2. Materials and methods

In this section, we will first describe the datasets and then discuss the methodology we used for collecting and analyzing the policies.

### 2.1. Data collection

The policy document data was extracted from the PKULaw Database. The PKULaw Database^1^ is the largest and most influential legal document search system that compiles public policies, laws, and regulatory documents in mainland China since 1949. The retrieval strategy was to obtain relevant policy documents using keywords, including “Rare Disease” (HANJIANBING) or “Orphan Drugs” (GUERYAO). Rare disease drugs were first mentioned in The Measures for Administration of Drug Registration, which was issued by former China Food and Drug administration in 2007 and repealed in 2020. A new version was then issued by State Administration for Market Regulation. The policy proposed special approval for new drugs with obvious clinical efficacy in the treatment of AIDS, malignant tumors, rare diseases, etc. Since 2009, China has begun to implement a series of new medical reform measures. Since then, the government has gradually focused on rare diseases. Therefore, the search of the study spanned from 2009 to 2022. The collection included documents at both the national and local government levels. However, since the content of local rare diseases policies was mainly based on national policies, only national-level policy documents were included in the analysis to obtain representative and effective national policies about China’s rare diseases. The official government websites of the central government and affiliated agencies, including the State Council, the National Health Commission, the National Healthcare Security Administration, and other relevant ministries and commissions, were used as supplementary databases to corroborate and supplement the policy documents. A full-text retrieval was performed on December 1st, 2022.

To guarantee accuracy and representation, the following criteria were established for filtering the policies chosen for this study. (1) Only current and valid policy documents were selected, and those that had been repealed, modified, or were in draft form were excluded. (2) Policies were chosen from laws, regulations, plans, opinions, notices, and measures that clearly and fully express the government’s intentions. Documents that only contained work summaries or information disclosures were not considered. (3) Policies that were not related to rare diseases or had only brief references without any substantial content were also excluded. It is worth mentioning that the policy screening was conducted by each of the three authors and any discrepancies were resolved through discussion and confirmation. After removing duplicates and irrelevant policies, a total of 61 valid policy documents on rare diseases were included in the study. Each selected policy document includes information on the policy-making departments, the date, and content related to rare diseases.

### 2.2. Analytical framework

**Figure 1:**
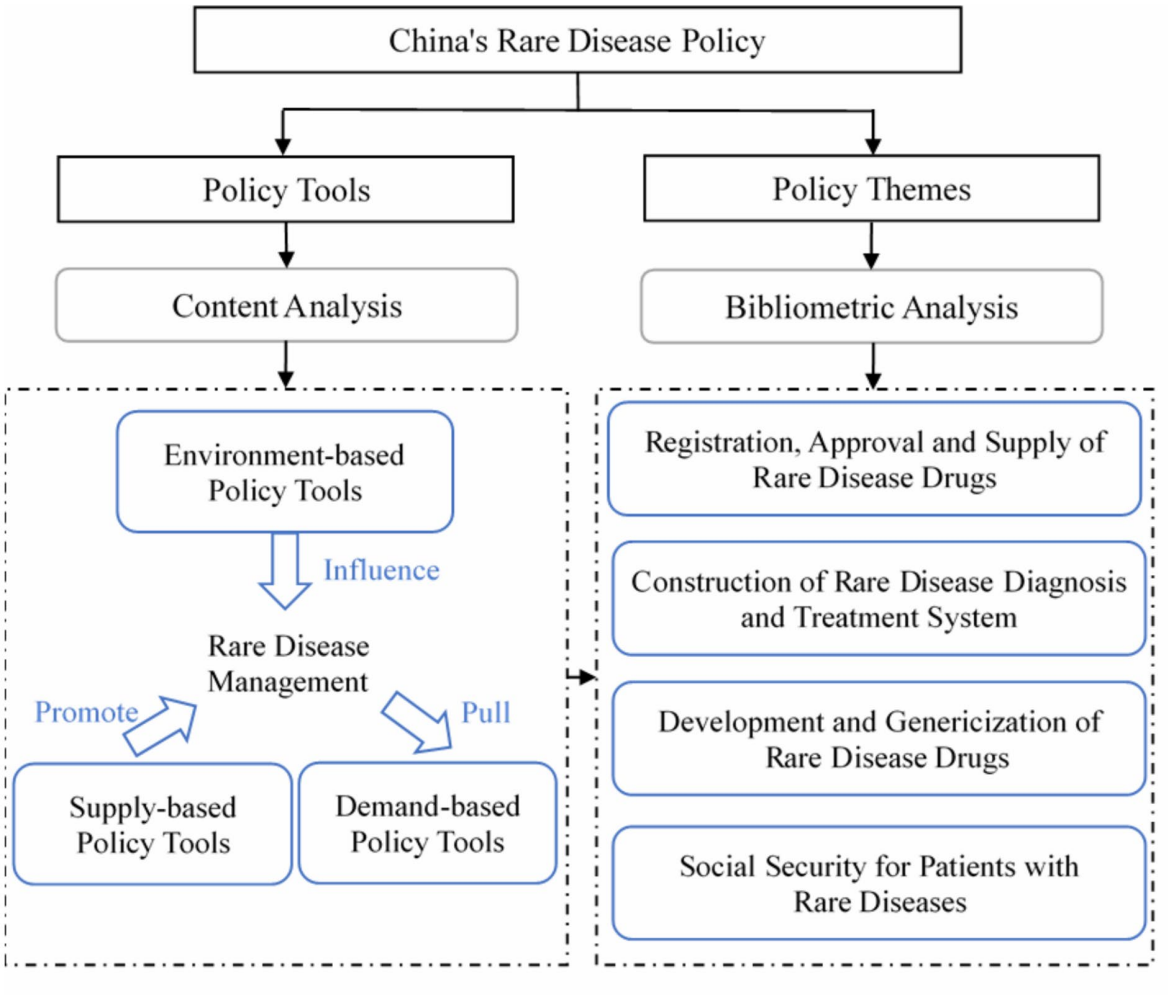
Research framework of policy for rare diseases in China.

This study adopts a mixed-methods approach that incorporates both content analysis and bibliometric analysis. We examine the metadata of rare disease policies, such as the date and policymakers, to analyze the temporal trends and identify the government agencies primarily involved in policymaking and those that collaborate more in the process. To quantitatively analyze China’s rare disease policy, a two-dimensional framework of “tools-themes” has been constructed, with the primary focus on analyzing policy tools and a complementary analysis of policy themes. The framework aims to examine the distribution of policy tools and themes and the use of specific tools for each policy theme, as shown in Figure 1.

#### 2.2.1. X dimension: the policy tool

Policy design is typically defined as the purposeful action of connecting policy tools with clearly stated policy goals (21). Policy tools refer to the government’s process of transforming its policy goals into a series of actions and mechanisms (22). The choice of policy tools has a significant impact on the success of achieving stated policy objectives and resolving potential or apparent policy problems in the state and social governance process (23). Scholars have developed various theoretical models of policy tools based on different classification perspectives. Rothwell and Zegveld’s classification (24) is still the most widely accepted in the literature on policy tools and remains the most commonly used in practical settings (25). They categorize policy tools into three types: environment-based, supply-based, and demand-based. This division aims to weaken the compulsory characteristics of the policy tools themselves and instead focus on the specific areas where the policy functions, thereby enhancing the relevance and content orientation of the policy tools (26). For this reason, this study adopts Rothwell and Zegveld’s classification to analyze the policy tools for rare diseases in China.

Environment-based policy tools work to establish a favorable social environment for the development of rare disease drugs and medical services by enhancing laws, regulations, and other public policies. Supply-based policy tools encompass the government’s efforts to improve the supply-side reform of rare disease drugs and medical services through the provision of human, financial, informational, technological, and other necessary resources. Demand-based policy tools address the needs of rare disease patients by exploring their demands through government procurement and medical insurance payment programs, among other initiatives. The classification and definition of these policy tools are displayed in Table 1.

**Table 1:** Classification and definition of policy tools.

Classification	Sub-classification	Definition
Environment-based policy tools	Goal planning	The development direction of rare disease policy is defined by formulating planning of objectives and tasks.
	Strategic measures	Various strategic measures, such as encouraging innovation and technology introduction, have been developed by the government to help improve the treatment, medication and security of patients with rare diseases.
	Regulation controls	The government enacts a series of laws and regulations to restrict or maintain the service behavior of hospitals and pharmaceutical companies.
	Tax incentives	The government provides tax exemptions or benefits to pharmaceutical companies engaged in the research and development, production and distribution of rare drugs.
	Standards and specifications	The government sets standards and specifications for issues related to the treatment, use of medication and security of patients with rare diseases.
	Performance evaluation	Evaluating achievements and rewarding for high performance.
	Intellectual property protection	The government strengthens IP protection for rare disease drugs.
Supply-based policy tools	Financial investment	The government provides direct financial assistance and financial support for rare disease drug development and other aspects.
	Information support	Build related databases and information network and make full use of information technologies to provide information exchange and information services to doctors and patients.
	Education and training	The government conducts various educational and training activities for the diagnosis and treatment of rare diseases and provides learning resources to improve the diagnosis and treatment capabilities of doctors.
	Organization construction	The government provides the necessary resources and services for the treatment, medication and security of rare disease patients by establishing and improving rare disease related organizations.
	Priority review and approval	Priority review and approval of rare drugs is an important part of public service, and the government gives priority to rare drugs in the drug review and approval process to promote rare drugs to market.
	Technical guidance	The government issues guidelines and standards for rare disease treatment and drug development, and organizes expert teams to guide rare disease management.
Demand-based policy tools	Government procurement	The government uses public funds to centralize procurement of rare disease drugs
	Medical insurance payment	The government includes of rare disease drugs in health insurance to promote access to rare disease drugs and services.
	Social support	The government and social forces provide social support to patients with rare diseases to meet their needs for basic living, education, mental health, social integration, etc.
	Public-private partnerships	The government cooperates with insurance companies or social forces to provide insurance products and public services for patients with rare diseases.
	International exchange	Encouraging medical institutions and pharmaceutical companies to carry out scientific research cooperation related to rare diseases with other countries and regions.

#### 2.2.2. Y dimension: the policy theme

Policy themes are reflections of the significant concerns derived from policy documents (27). This study utilized policy themes as the Y dimension in its policy analysis framework. Bibliometric analysis techniques, such as co-word analysis and cluster analysis, are commonly used in mapping research themes within policy (28–31). This study conducted theme extraction and visualization analysis of rare disease policies through the use of co-word analysis and cluster analysis. The process consisted of three main steps: (1) Word segmentation and word frequency statistics were performed using Jieba and Pandas for Python on all rare disease policy texts, resulting in the identification of high-frequency words and their document distribution. (2) A standardized keyword list was created through manual screening, cleaning, and merging of synonyms of the high-frequency words to accurately reflect the content of rare disease policies. (3) A co-occurrence matrix for the cleaned high-frequency words was constructed using Python, and the co-occurrence relationship was visually displayed and clustered by Gephi. This divided the co-occurrence network of keywords into multiple subgroups, enabling the identification of themes within rare disease policies (32, 33).

### 2.3. Policy documents content coding

The policies were treated as the basic units of analysis in this study and were systematically coded and extracted from the collected policy documents. The format of the coded policies was “policy number-chapter number-section number-entry number.” After numbering the completed policy documents, manual coding was carried out based on Rothwell and Zegveld’s classification of policy tools and policy themes. The coding results were analyzed using Excel 2021, with a content analysis performed on the two-dimensional framework. To ensure the high reliability and consistency of the coding, prior to initiating coding, the coding personnel were first familiarized with the meaning of each policy instrument to fully understand the coding criteria. Coding results were compared between two independent coders using the index of category agreement (CA). The formula for calculating the index of category agreement is as follows: $CA=\frac{2S}{T1+T2}$ , where T1 and T2 denotes the number of codes for each of two coders, respectively, and S is the number of consistent codes derived from the two coders. The calculated result was 0.928, showing that the two coders have high coding consistency and stability. For inconsistent coding data, an expert intervened to discuss and settle the controversial part through negotiation.

## 3. Results

### 3.1. Temporal distributions

**Figure 2:**
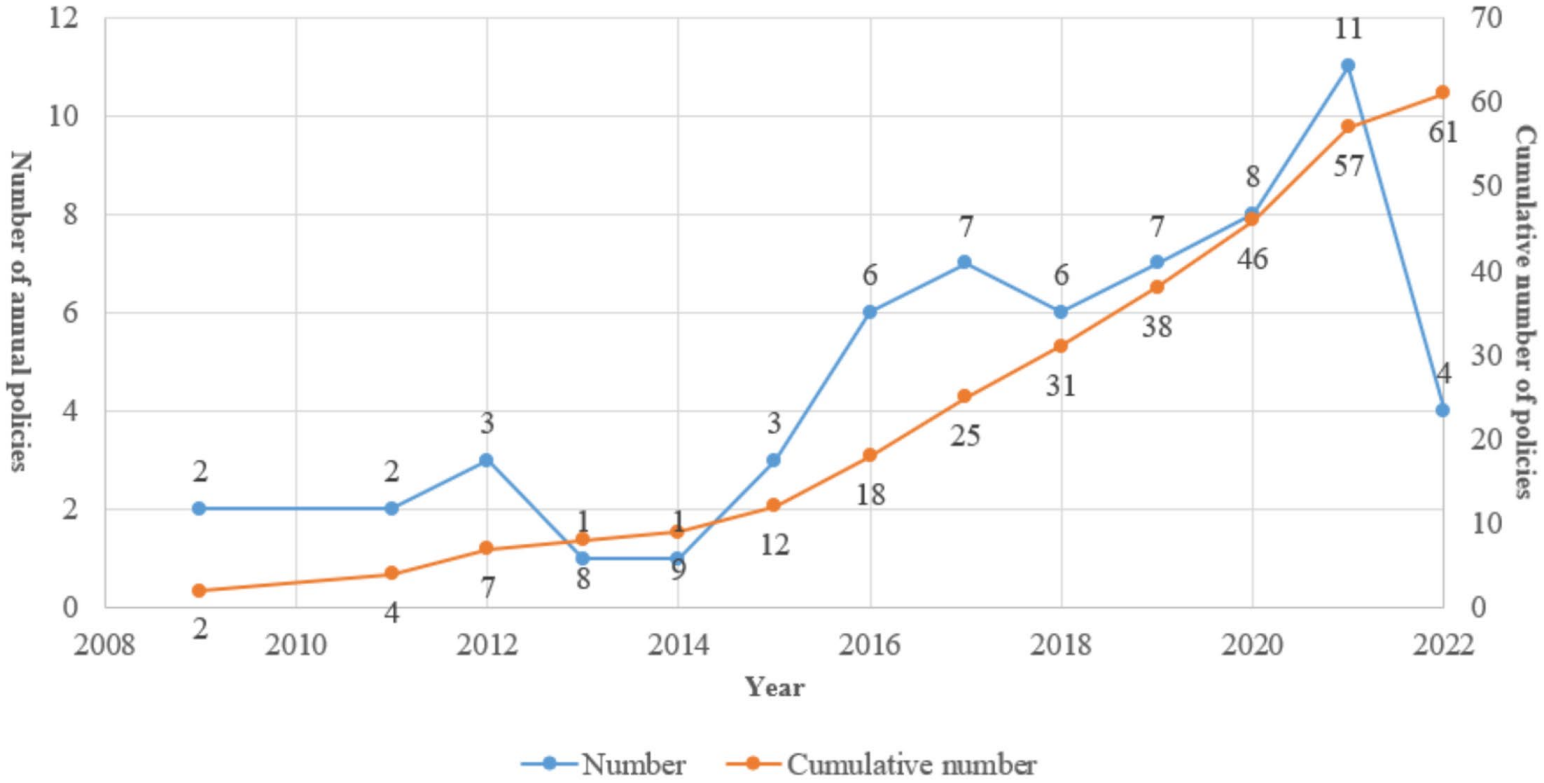
Time distribution of China’s rare disease policies, 2009–2022.

From the description in Figure 2, it is clear that the Chinese government has made significant progress in addressing the issue of rare diseases. The number of policies related to rare diseases has increased dramatically since 2016, with the highest number of policies being issued in 2019. The release of the First National Rare Disease List in 2018 has also played a crucial role in bringing awareness to the issue and has facilitated public communication, patient services, and research studies in the field of rare diseases (34). However, it is also important to note that the field of rare disease policy is still in its early stages in China, and there is still much work to be done in order to fully support those affected by rare diseases. The government will likely continue to issue policies and take action to support the rare disease community in the coming years.

### 3.2. Policymakers

The information in Table 2 highlights the standardization of the names of policymakers and the departments that issued rare disease policies. The standardization was based on the State Council Institutional Reform Plan of March 2018 and took into account the operations of the departments at the time of policy issuance. Of the 61 rare disease policies, 47 (77.05%) were issued independently by one department, while 14 (22.95%) were issued jointly by several departments. This shows that some rare disease policies were developed and implemented in a collaborative effort between multiple government agencies, which could indicate a coordinated approach to addressing the issue of rare diseases. Overall, the involvement of multiple departments in the formulation of rare disease policies provides a clearer understanding of the government’s efforts to address the issue of rare diseases in China. In addition, the bureaucratic relationships and main functions of policy makers were shown in Supplementary Table.

**Table 2:** Statistics of rare disease policy-issuing department.

Policymakers	Abbreviations for policymakers	Number of policies	Number of independent policies	Number of joint policies
National health commission	NHC	17	12	5
State council	SC	16	13	3
National medical products administration	NMPA	14	9	5
Ministry of science and technology	MOST	4	1	3
Ministry of human resources and social security	MOHRSS	4	1	3
National healthcare security administration	NHSA	3	0	3
National administration of traditional chinese medicine	SATCM	3	0	3
Central committee of the communist party of china	CCOCPC	3	0	3
State administration for market regulation	SAMR	3	3	0
Ministry of civil affairs	MCA	3	1	2
Ministry of industry and information technology	MIIT	2	2	0
Standing committee of the national people’s congress	SCONPC	2	2	0
China banking and insurance regulatory commission	CBIRC	2	2	0
Ministry of finance	MOF	2	0	2
Logistical support department of central military commission	LSDOCMC	2	0	2
China disabled persons’ federation	CDPF	1	1	0
National development and reform commission	NDRC	1	0	1
General administration of sport	GAOS	1	0	1
General administration of customs	GAOC	1	0	1
Ministry of education	MOE	1	0	1
State taxation administration	STA	1	0	1
Chinese academy of sciences	CAS	1	0	1
Chinese academy of engineering	CAE	1	0	1
National natural science foundation	NSFC	1	0	1

The top 3 government agencies involved in the development of rare disease policies in China are the National Health Commission, the State Council, and the National Medical Products Administration. The National Health Commission was responsible for 17 policies, the State Council for 16 policies, and the National Medical Products Administration for 14 policies. It is noteworthy that the State Council independently issued 13 policies, while jointly issuing 3 policies in collaboration with other departments. The National Health Commission independently issued 12 policies, while jointly issuing 5 policies with other departments. The National Medical Products Administration independently issued 9 policies, while jointly issuing 5 policies with other departments.

**Figure 3:**
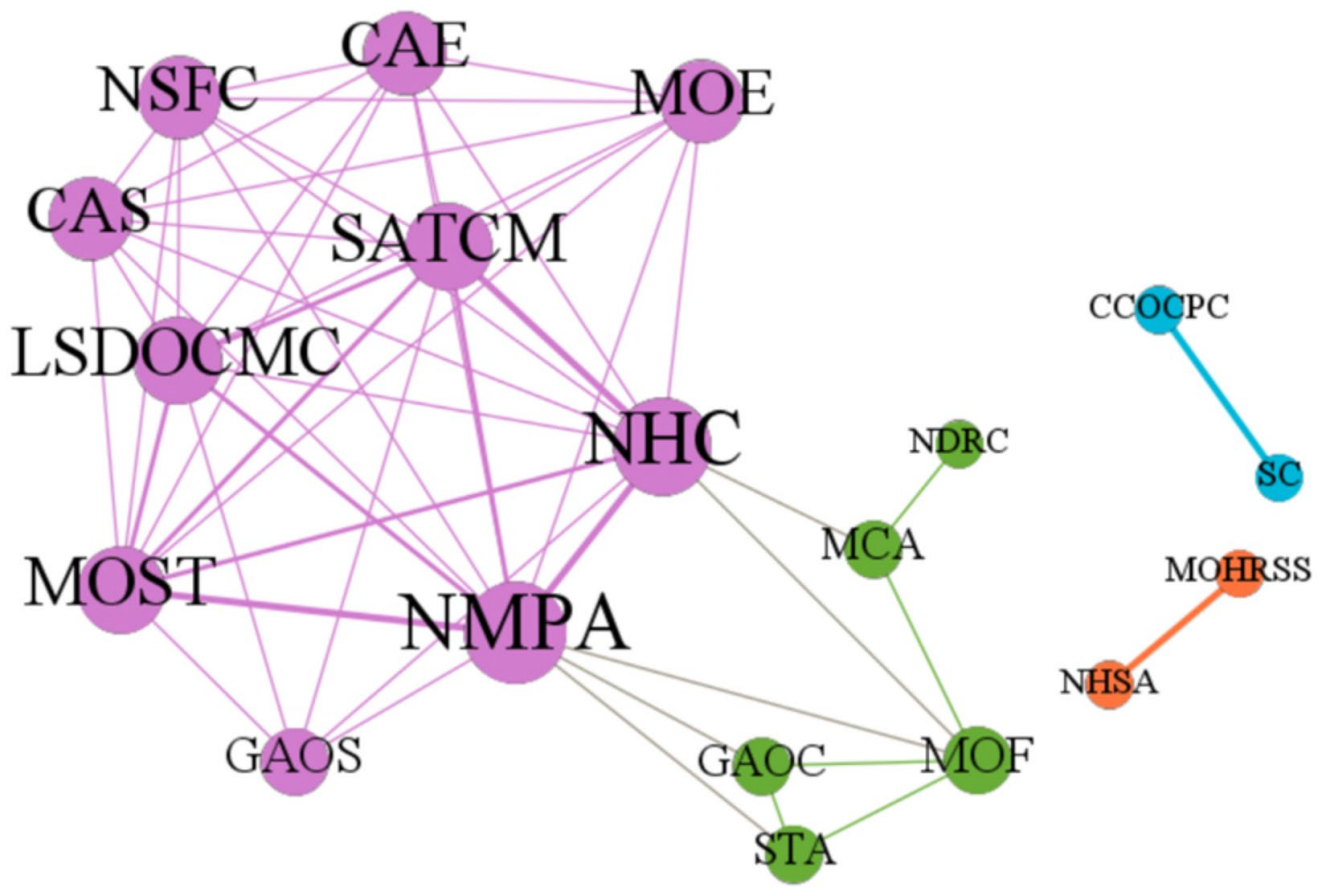
Network of rare disease policy-making departments in China.

Most of the 14 policies jointly issued by the policymakers are plans and guidelines. As depicted in Figure 3, the collaborative network of the policymakers who issued the rare disease policies was created using Gephi software. The size of the nodes signifies the policymakers’ degree of centrality, the thickness of the lines represents their cooperation frequency, and the node color signifies the results of the Gephi modularity algorithm’s community grouping. In the departmental cooperation network, it can be observed that a main circle of decision-makers, represented by the purple nodes, has formed with the core participation of the National Health Commission, the National Medical Products Administration, the National Administration of Traditional Chinese Medicine, the Ministry of Science and Technology, and other relevant government departments with supplementary participation. These departments are closely connected and collaborate to issue policies on the development and registration of rare disease drugs. The green nodes are located on the periphery of the main collaborative network and are comprised of the Ministry of Finance, the State Taxation Administration, and the General Administration of Customs, along with the National Medical Products Administration. These agencies are responsible for policies on the import of rare disease drugs. The Ministry of Finance, the Ministry of Civil Affairs, and the National Health Commission are responsible for policies related to patient assistance with rare diseases. The adjustment of the tax rate for imported rare disease drugs and the implementation of relief measures for patients are dependent on the guidance and support of the Ministry of Finance. The State Council and the Central Committee of the Communist Party of China, represented by the blue nodes, jointly issued three policies. Another closely collaborating pair of agencies is the National Healthcare Security Administration and the Ministry of Human Resources and Social Security, represented by the orange nodes, which jointly publish the National Health Insurance Medicine Catalogue that includes drugs for rare diseases.

### 3.3. Analysis of policy tools

Table 3 presents the frequency and proportion of each policy tool and its sub-components. The results indicate that environment-based policy tools are the most frequently utilized, accounting for 48.08% (50 items in total). Supply-based policy tools were the second most commonly used, accounting for 38.46% (40 items in total), while demand-based policy tools were the least frequently utilized, accounting for only 13.46% (14 items in total).

**Table 3:** The frequency and proportion of policy tools.

Classification	Sub-classification	Frequency	Proportion
Environment-based policy tools (50, 48.08%)*	Strategic measures	22	21.15%
	Regulation controls	11	10.58%
	Goal planning	7	6.73%
	Standards and specifications	5	4.18%
	Performance evaluation	2	1.92%
	Intellectual property protection	2	1.92%
	Tax incentives	1	0.96%
Supply-based policy tools (40, 38.46%)	Priority review and approval	12	11.54%
	Technical guidance	10	9.62%
	Information support	8	7.96%
	Organization construction	6	5.77%
	Education and training	2	1.92%
	Financial investment	2	1.92%
Demand-based policy tools (14,13.46%)	Medical insurance payment	6	5.77%
	Social support	4	3.85%
	Public-private partnerships	2	1.92%
	Government procurement	1	0.96%
	International exchange	1	0.96%

* The number in the bracket is the frequency of each tool with its proportion followed.

In terms of the components of environment-based policy tools, strategic measures were the most frequently used, accounting for 21.15% (22 items in total), followed by regulation controls (10.58%, 11 items in total), goal planning (6.73%, 7 items in total), and standards and specifications (4.18%, 5 items in total). The usage of performance evaluation and intellectual property protection was low, accounting for 1.92% (2 items in total) and 1.92% (2 items in total), respectively. Tax incentives were used only once, accounting for 0.96% (1 item in total).

For the supply-based policy tools, the highest frequency of use was for priority review and approval (11.54%, 12 items in total) and technical guidance (9.62%, 10 items in total). Information support and organization construction were used, respectively, 8 and 6 times, accounting for 7.96 and 5.77% of the total. Financial investment as well as education and training were the least utilized, accounting for 1.92% (2 items in total) each.

Demand-based policy tools were underutilized. Among the demand-based policy tools, medical insurance payment and social support were used the most, accounting for 5.77% (6 items in total) and 3.85% (4 items in total), respectively. Public-private partnerships, government procurement, and international exchange were used only 2, 1, and 1 times, respectively, accounting for 1.92, 0.96, and 0.96% of the total.

### 3.4. Analysis of policy themes

**Figure 4:**
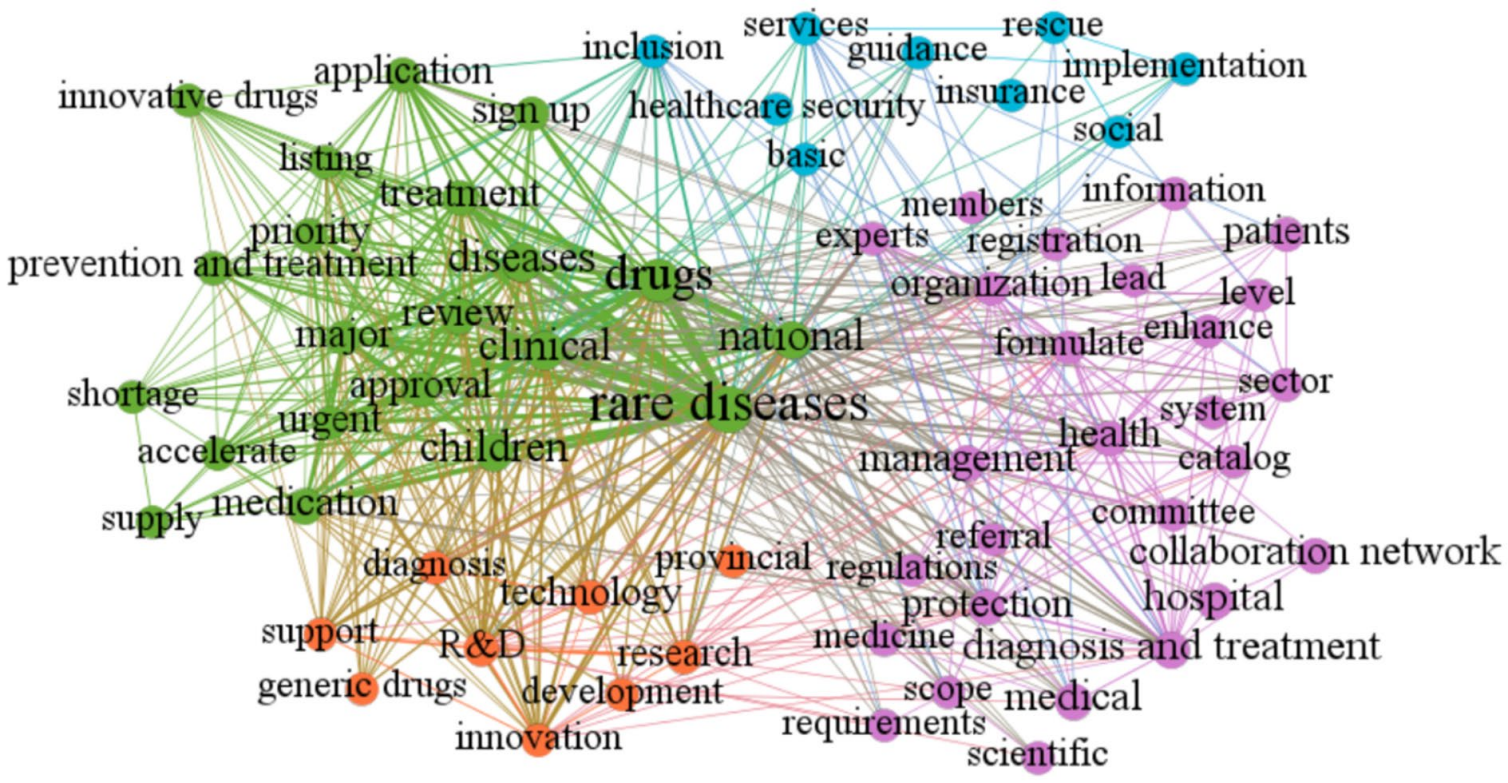
High-frequency word network of rare disease policies during 2009–2022 in China.

The Gephi software was employed to construct a clustering map that aimed to uncover the thematic distribution of policies related to rare diseases. The visualization network comprised of nodes, the size of which represented the frequency of thematic words, and lines that indicated the intensity of word co-occurrence. The different clusters (themes) were indicated by different colors. After tracing the source of high-frequency thematic words and reviewing relevant policy texts, the rare disease policy themes were organized into four main categories (as illustrated in Figure 4).

Registration, Approval, and Supply of Rare Disease Drugs: This category is represented by the green clustering and encompasses keywords such as “approval,”“review,”“listing,” and “supply.” It highlights the importance of this area in rare disease policy.

Construction of Rare Disease Diagnosis and Treatment System: This category is indicated by the purple clustering and encompasses policy measures aimed at diagnosing and treating rare diseases. Keywords in this category include “diagnosis and treatment,”“hospital,”“collaboration network,” “registration,” and “information.”

Development and Genericization of Rare Disease Drugs: This category, represented by the small orange node, highlights that rare disease drug research and development in China is still in its infancy. Keywords in this category include “R&D,” “development,” “innovation,” “generic drugs,” and “technology.”

Social Security for Patients with Rare Diseases: This category is indicated by the blue clustering and encompasses social security policies for individuals with rare diseases. Keywords in this category include “healthcare security,” “insurance,” and “rescue.”

### 3.5. Two-dimensional analysis of policy tools -policy themes

**Figure 5:**
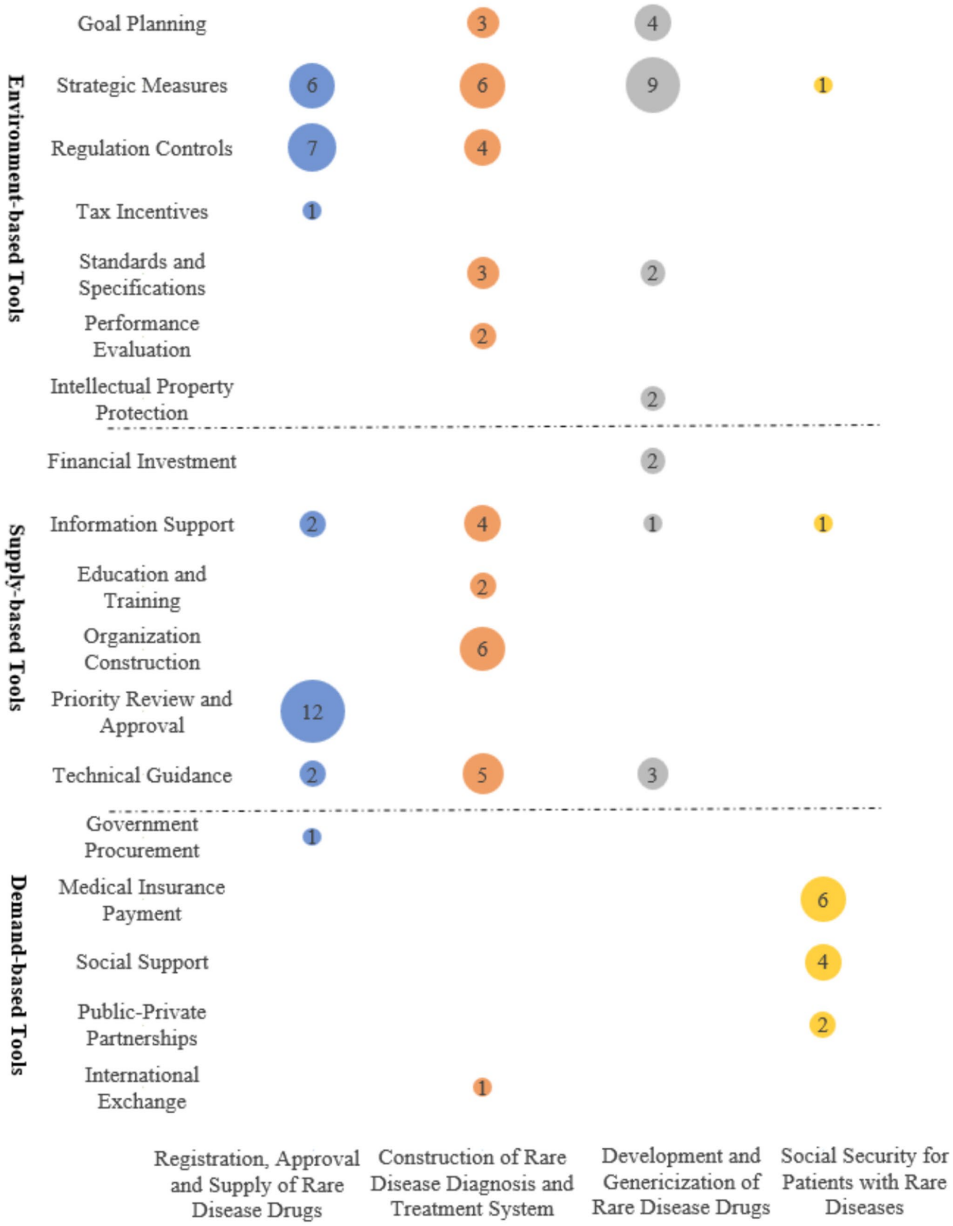
Policy “Tools-Themes” two-dimensional distribution.

This study maps the policy themes identified to policy tools. Figure 5 displays the distribution of these three types of policy tools in each policy theme. The Registration, Approval and Supply of Rare Disease Drugs employs 16 supply-based, 14 environment-based, and 1 demand-based tools, with priority review and approval being the most frequently applied, accounting for 38.71% (12 items in total). The Construction of the Rare Disease Diagnosis and Treatment System involves the use of 17 supply-based, 18 environment-based, and 1 demand-based tools, with strategic measures and organization construction being the most frequently applied, accounting for 16.67% (6 items in total) each. The Development and Genericization of Rare Disease Drugs employs 6 supply-based and 17 environment-based tools, with strategic measures being the most frequently applied at 38.17% (9 items in total). For Social Security for Patients with Rare Diseases, environment-based tools are applied most often, with 12 items, while supply-based and demand-based tools each have 1 item. Medical insurance payment tools are the most frequently used, accounting for 42.86% (6 items in total).

## 4. Discussion

### 4.1. Principal findings

Policy-making departments have embedded their values in the content of policies and the tendency to use policy tools. For China, even a disease with a small chance of occurrence is a huge number in the face of a population of 1.4 billion. It is the orientation of the Chinese government to respect and protect the rights of people with rare diseases. Prior to the 2009 medical reform, there was only one policy that addressed rare disease drugs. However, since then, the number of rare disease policies has significantly increased, demonstrating China’s commitment to addressing this issue. Currently, the rare disease policies are developed in the context of the disease situation of our population, the level of medical technology, and the burden of disease. The National Health Commission, the State Council, and the National Medical Products Administration have taken the lead in formulating most policies and collaborating with other departments. In China, orphan drugs are undersupplied. According to statistics, as of December 2018, there are 162 therapeutic drugs for a total of 74 rare diseases out of 121 rare diseases in the First National Rare Disease List. However, there are 79 drugs outside the country but not listed in the country, involving 21 diseases. There are still 35 listed drugs that have no indications from the list (13). Since China’s ability to develop innovative drugs is less than that of developed countries such as Europe, America and Japan, the primary concern for China is how to access existing drugs for rare diseases. So, policy themes show a related tendency, such as the use of accelerated review policies to speed up the import of foreign rare disease drugs already on the market, as well as to encourage Chinese pharmaceutical companies to develop rare disease drugs. Another characteristic of China is that due to geographical factors and the level of economic development, patients across different regions and between urban and rural areas have different medical resources and security systems. Therefore, the government’s commitment to improve the level of rare disease treatment and patient protection is to give rare disease patients equal economic, social and treatment rights. The policy tools for rare diseases primarily emphasize supply and environment-based solutions. While some policy tools have supported the development of rare disease management, others require adjustment to be more effective.

### 4.2. Improving the top-level design of rare disease policy

Overall, the formulation of rare disease policies in China is primarily led by multiple departments. The State Council, the country’s highest administrative body, issued independent, macroscopic and instructive policies (31), mainly providing guidance and recommendations for the development of rare disease policies. Meanwhile, government affiliates typically draft specific measures in response to the central government’s directives. For example, the National Health Commission is responsible for the prevention and treatment of rare diseases, the National Medical Products Administration manages the registration and supervision of rare disease drugs, and the National Healthcare Security Administration oversees medical coverage for rare diseases. While various government departments are becoming more involved in rare disease policy-making, it’s crucial to avoid policy fragmentation. Notably, most policies are issued independently by individual departments, indicating a potential lack of coordination and cooperation mechanisms in China’s rare disease field.

Managing rare diseases is a complex undertaking that requires a comprehensive systems approach. Several countries have already developed national action plans or strategies for rare diseases (35–38) that involve collaboration between various departments and are led by the healthcare administration. These strategies aim to establish a multifaceted system to ensure the prevention, treatment, research, medication, information management, education, and social support of rare diseases. However, China currently lacks a complete and unified strategy for rare diseases. In the future, rare disease management should be treated as a national strategy, and the objectives of rare disease policies should be clearly defined. The government should continuously adjust and optimize the policy objectives and implementation process around rare diseases, enhance effective coordination between multiple departments, and improve the overall rare disease policy system.

### 4.3. Imbalance in the use of policy tools

#### 4.3.1. The using of environment-based policy tools

Environment-based policy tools currently dominate rare disease policies in China. Strategic measures, such as national encouragement and support for rare disease treatment and drug development, are employed in environment-based policies. Since 2019, revisions to the Drug Administration Law and the Measures for Administration of Drug Registration have further clarified the registration of rare disease drugs and the procedures and contents of rare disease management. While the performance evaluation tool is used in the Rare Disease Diagnosis and Treatment Collaborative Network, this policy tool needs improvement to provide a scientific performance system to assess the quality of care for rare diseases.

Intellectual property (IP) protection and tax incentives tools are relatively few in number, which has failed to harness the full potential of pharmaceutical companies. In other countries, government R&D grants, tax breaks, and market exclusivity policies have been powerful incentives for pharmaceutical companies to develop drugs for rare diseases. For example, the U.S. offers tax deductions for clinical research costs and tax relief for orphan drugs, while exempting applicants for orphan drugs from FDA review fees and granting a 7-year market monopoly period (14). A series of incentives implemented in the U.S. has greatly facilitated the innovation and development of rare disease drugs and has led the world in rare disease drug development to date. Some experts believe that legislation of an orphan drug act in China would remarkably accelerate orphan drug development and potentially lower costs (39). In China, the valued added tax (VAT) rate for imported drugs to treat rare diseases has been reduced to 3%. Some pharmaceutical companies engaged in the R&D of rare disease drugs can be recognized as high-tech enterprises and benefit from preferential corporate income tax policies. IP protection policies are relatively uncommon in China, but are an important environment-based policy tool to encourage rare disease drug development and one that the Chinese government is working to improve. The new Patent Law in 2020 added a patent term compensation system and a drug patent linkage system, reflecting China’s efforts in drug patent protection. In May 2022, National Medical Products Administration issued the “Regulations on the Implementation of the Drug Administration Law of the People’s Republic of China (Draft Revision for Public Comments) “, which provided a special policy of “market exclusivity” for rare disease drugs, stipulating that new drugs approved for marketing for rare diseases can be granted a maximum of 7 years of market exclusivity. This is the first time that the market exclusivity period of rare disease drugs is included in the regulations. The regulation also provides a special chapter on drug intellectual property protection, including patent linkage, promotion of generic development and data protection, together with the market exclusivity system, which builds an enhanced drug IP protection network. Once the regulation is introduced, it will greatly stimulate the innovation of new drugs for rare diseases in China.

There is currently a lack of financial support tools in rare disease policies. The long R&D cycle of new rare disease drugs requires large investments and constant financing to ensure normal development. To leverage the capital market’s support for innovative pharmaceutical companies, several of China’s stock exchanges have created new segments, for example, the Shanghai Stock Exchange established the Science and Technology Innovation Board, the Shenzhen Stock Exchange pioneered the Small and Medium Enterprises segment, and the Beijing Stock Exchange was established, all of which provide venues for pre-revenue pharmaceutical companies to raise financial capital to fund drug development including those for rare disease. In addition, Hong Kong Stock Exchange also established similar policy years earlier.

Additionally, environment-based policy tools such as public education can be used to increase awareness of rare diseases and encourage the public to support patients and families affected by rare diseases.

#### 4.3.2. The using of supply-based policy tools

The most widely used supply-based tool for drug approval is the priority review and approval process, which is also the cornerstone of China’s rare disease policy. Prior to 2016, policies primarily focused on the registration, review, and approval of rare disease drugs. However, in 2018, China issued the “Announcement on Matters Relating to the Review and Approval of Foreign New Drugs in Urgent Clinical Need,” which established a special channel for the review and approval of new drugs in urgent clinical need from abroad. Since then, three batches of the List of Foreign New Drugs in Urgent Clinical Need have been released, with 41 drugs approved for the treatment of rare diseases. In 2019, the Drug Administration Law was revised, formally establishing a priority review and approval system for rare disease drugs at the legal level. The revised Drug Registration Management Measures in 2020 specified that innovative and improved new drugs for rare diseases with obvious clinical value would be included in the priority review and approval process, with a time limit of 70 days for rare disease drugs that have been approved abroad but not yet in China. As of 2021, 33 rare disease drugs (3 drugs in 2018, 6 drugs in 2019, 11 drugs in 2020, and 13 drugs in 2021) (40, 41) have been approved for listing through the priority review and approval process, covering over a dozen rare diseases such as Spinal Muscular Atrophy, Multiple Sclerosis, and hereditary angioedema. By the end of 2021, based on the First National Rare Disease List, China has marketed 150 drugs for 103 rare diseases (42), effectively solving the problem of drug unavailability for rare disease patients. However, China does not have a separate qualification process for rare disease drugs, instead, they are declared as a type in the priority review and approval process. In the future, a dedicated national rare disease drug management agency should be considered.

In addition to the priority review and approval process, the Chinese government also emphasizes the importance of technical guidance as a supply-based policy tool for rare disease treatment. In China, it is commonly said that “physicians who treat rare diseases are rarer than patients with rare diseases,” reflecting the current situation of rare disease treatment. An online survey conducted in 2018, which included 2040 patients with rare diseases in China, showed that more than two-thirds had experienced misdiagnosis (43). To address this issue, the National Health Commission established an expert committee on rare disease treatment and security in 2015, with the aim of defining rare diseases in line with China’s national conditions, developing technical specifications and clinical pathways for rare disease prevention and treatment, and providing advice on prevention, screening, treatment, medication, and security. The Rare Diseases Diagnosis and Treatment Guidelines were subsequently published by the National Health Commission in 2019. To further encourage pharmaceutical companies to carry out research and development of rare disease drugs and improve the efficiency and quality of clinical trials, the National Medical Products Administration issued two technical guidelines for rare diseases in 2021 and 2022. These guidelines include the “Technical Guidelines for Clinical Development of Drugs for Rare Diseases” and the “Guidelines on Statistics for Clinical Research of Drugs for Rare Disease (for Trial Implementation),” which provide recommendations based on the unique characteristics of rare diseases.

Information support tools mainly refer to the construction of a rare disease registration platform. The establishment of such a platform can form a rare disease knowledge base, realize multi-level sharing of rare disease data, reduce the time for physicians to diagnose, and improve the accuracy of diagnosis (44). Organization construction tools mainly refer to the Rare Disease Diagnosis and Treatment Collaborative Network, which covers 324 hospitals. The network has established a two-way referral model and teleconsultation system, and carries out direct reporting of rare disease cases. To further improve the field of rare disease treatment, the government should continue to increase the supply of human, material, financial, information, and public services. For example, rare disease topics should be incorporated into medical school curriculums, and scientific and effective training plans should be formulated to ensure that doctors are adequately trained in the field of rare diseases (39). Adequate financial investment is a key element in promoting research and development of rare disease drugs and improving the diagnosis and treatment of rare diseases. The government should continue to increase financial support for the field of rare diseases to ensure that rare disease patients have access to the best possible treatments.

#### 4.3.3. The using of demand-based policy tools

Demand-based policy tools account for a small percentage (13.46%) of all policy tools. The current policy landscape regarding rare disease drug use and treatment in China is primarily driven by medical insurance payment measures. However, progress has been made in recent years. In 2012, the new rural cooperative medical care program prioritized the inclusion of 12 rare diseases, including hemophilia, in pilot coverage for critical illness insurance. Since then, the number of rare disease drugs included in the national medical insurance catalogue has increased, and some drug prices have been significantly reduced. In fact, from 2017 to 2021, the medical insurance catalogue underwent four adjustments, and a total of 62 rare disease drugs were added to the coverage list. For those rare disease drugs excluded from the national medical insurance catalogue, some provinces and cities in China have introduced rare disease drug coverage models with local characteristics (14). Qingdao’s supplementary medical insurance, established a special drug catalog, including drugs for the treatment of hemophilia, hyperphenylalaninemia, Gaucher disease, etc. Zhejiang Province established a special fund for rare disease drugs to guarantee medication for patients with Gaucher disease, Fabry disease, type II glycogen storage disease and phenylketonuria since 2019. Jiangsu Province has also adopted a special fund model for rare diseases since 2021. Drugs used to treat Phenylketonuria, Gaucher disease, Pompe disease, and Fabry disease were included in the health insurance drug catalog for severe diseases in Shandong Province in 2020. Foshan City has included all 121 rare diseases from the First National Rare Disease List in the scope of medical aid.

Given the limited government medical insurance fund, additional security models such as commercial insurance are critical to addressing the high costs associated with rare disease treatments. Therefore, the government should consider strengthening cooperation with commercial insurance companies to develop insurance products that can benefit rare disease patients. Additionally, special arrangements for procuring drugs for the treatment of rare diseases should be considered in future policies.

Furthermore, enhancing international cooperation in the field of rare disease response, such as through sharing information, resources, and collaborating on research projects, can also improve the national capacity to respond to rare diseases. Overall, continued efforts and collaboration among various stakeholders are necessary to further advance policies and support for rare disease patients in China.

### 4.4. Adjusting the focus of policy themes

China’s rare disease policy system has made progress in recent years, but there are still gaps in the supply of policies for different themes. The two main themes that require attention are the Construction of Rare Disease Diagnosis and Treatment System and the Registration, Approval, and Supply of Rare Disease Drugs. However, the Development and Genericization of Rare Disease Drugs, as well as Social Security for Rare Disease Patients, remain relatively insufficient.

Many planning and guiding policies have addressed rare disease drug development, including the “Notice of the State Council on National Drug Safety During the 12th Five-Year Plan,” the “13th Five-Year Plan for Health,” and the “14th Five-Year Plan for the Development of Pharmaceutical Industry.” These documents have included rare disease drugs as a key support target, proposing to guide enterprises to strengthen the R&D of drugs and medical devices for rare diseases. Additionally, the “Opinions of the General Office of the State Council on Reforming and Improving Policies to Ensure the Supply and Govern the Use of Generic Drugs,” introduced in 2018, has encouraged pharmaceutical companies to produce generic versions of drugs necessary for clinical treatment, especially for the treatment of rare diseases. Despite progress, more national policies are needed to drive independent R&D, increase the variety and number of rare disease drugs, and provide more patients with access to treatment.

Rare diseases often have severe physical, psychosocial, and economic impacts on patients and their families. Diagnostic and treatment uncertainty exacerbate these disabilities. (45). Unfortunately, the affordability of rare disease drugs in China is not optimistic (14, 46). To address these challenges, China is exploring the establishment of a multi-level protection system led by national medical insurance and shared by commercial insurance, charity, and medical assistance. The positive exploration of medical coverage for rare diseases in Zhejiang, Jiangsu and Shandong will also provide practical models for the national coverage for rare diseases, which has a very important reference value. Based on the evaluation of the effectiveness of different security models, the government could consider their feasibility for national expansion. The national model of rare disease security should be constructed, taking into account the drug management system, patient composition, economic level and legal system. There is a need for a more scientific design of the sources and payment coverage and reimbursement rates for rare disease coverage funds. In addition, a comprehensive system of assistance for patients and families with rare diseases should be considered in policy planning. Furthermore, future policies should ensure the right to education and employment for people with rare diseases and provide mental health services to improve social integration.

In conclusion, while China’s rare disease policy system has made progress in recent years, there is still room for improvement in the development and distribution of policies related to rare disease drugs, social security for rare disease patients, and assistance for patients and families. National policies that drive independent R&D and provide more patients with access to treatment, as well as policies that address the broader social and economic impacts of rare diseases, are necessary to better support this vulnerable population.

### 4.5. Improving the coordination of policy tools and policy themes

The allocation of policy tools must be a well-thought-out process that takes into account policy objectives and themes. Unfortunately, policy tools for rare diseases are unevenly distributed across themes. Currently, more policy tools are being applied to the registration, approval, and supply of rare disease drugs, as well as the construction of rare disease diagnosis and treatment systems. However, the government primarily relies on environment-based and supply-based tools, with very few demand-based tools used in these areas. Conversely, social security for patients with rare diseases is dominated by demand-based tools.

To improve the rare disease policy system, the government must fully consider the coordination of policy tools and themes, and continuously adjust and optimize the proportion of environment-based, supply-based, and demand-based policy tools used, as well as their internal structure. Implementing demand-based tools, such as government procurement and medical insurance payments, can incentivize pharmaceutical companies to enhance the development and supply of drugs for rare diseases. Additionally, pilot demonstrations and international exchanges can enhance rare disease treatment capabilities. Given that rare diseases are a universal challenge for all countries in the world, maintaining an open attitude is crucial. This includes being open to research results, treatment experiences, and drugs from other countries, as there is much to learn, exchange, introduce, and gain from such efforts. To improve construction of rare disease diagnosis and treatment systems and social security for rare disease, the government should establish special funds and increase investment.

## 5. Conclusion

This article presents a study that aimed to identify the characteristics of rare disease policies in China using content analysis on policy documents and bibliometric analysis. The study explored policy tools and themes and provided insights that could guide the improvement and optimization of rare disease policies in China and other countries.

The study found that rare disease policies in China involve multiple sectors such as healthcare, social care, insurance, and education, among others. To foster a more equitable and inclusive community for the rare disease population, the government needs to introduce a unified rare disease action plan at the national level. The study also revealed that China’s rare disease policy mainly adopts environment policy tools to create a sustainable environment for the development and listing of rare disease drugs and diagnosis and treatment.

The study highlights the need to focus on supply-based and environment-based policy tools to strengthen the prevention, treatment and management of rare diseases. The results suggest the importance of strengthening the use of demand-based tools and optimizing the internal structure of supply-based and environment-based tools to promote the development and listing of rare disease drugs. The findings also suggest that the use of supply-based tools should be enhanced to provide multi-level social security for patients with rare diseases.

In this study, we demonstrate a new approach to policy analysis that is not limited to the analysis of public health policies, but can be extended to a variety of public policies. Using this approach, researchers and policy makers can both track the current state of public policy and the rationale for the use of policy tools, as well as inform public policy proposals.

The study acknowledges its limitations, including the bias toward national-level government agencies and the complexity of policy analysis. This approach of policy analysis can only objectively demonstrate the situation of policy themes and the use of policy tools, but their importance needs to be further investigated and analyzed. However, it provides valuable insights that can help policymakers in the management of rare diseases. Future research should focus on combining supply and demand analysis of policy tools, emphasizing the synergistic effects of different types of policy tools, and the policy impacts of different levels of government.

In summary, this article provides a comprehensive analysis of rare disease policies in China and highlights the importance of a coordinated approach involving multiple sectors and policy tools to address the needs of the rare disease population. The article offers valuable insights that can guide policymakers in improving rare disease policies in China and other countries.

## Data availability statement

The raw data supporting the conclusions of this article will be made available by the authors, without undue reservation.

## Ethics statement

This study is an analysis of publicly available policy documents. Therefore, the approval of the institutional review committee is not required.

## Author contributions

YM contributed to the design of the study. LY, MW, and YH collected data. XL, WL, and YM conducted the statistical analysis and wrote the first draft of the manuscript. All authors contributed to the article and approved the submitted version.

## Funding

This study was funded by the Subject of Humanities and Social Sciences of Shandong Province (2021-YYGL-28), Shandong First Medical University (Shandong Academy of Medical Sciences) 2022 Youth Science Foundation Incubation Grant Program (202202-001), the Fundamental Research Funds for the Central Universities (no ND2022016), the Innovation Project of Shandong Academy of Medical Sciences and Academic promotion program of Shandong First Medical University. There was no direct involvement by the funder in any aspects of the study.

## Conflict of interest

The authors declare that the research was conducted in the absence of any commercial or financial relationships that could be construed as a potential conflict of interest.

## Publisher’s note

All claims expressed in this article are solely those of the authors and do not necessarily represent those of their affiliated organizations, or those of the publisher, the editors and the reviewers. Any product that may be evaluated in this article, or claim that may be made by its manufacturer, is not guaranteed or endorsed by the publisher.

## Footnotes

^1^ http://www.pkulaw.cn
