# Supplementary material for: Policy analysis in the field of rare diseases in China: a combined study of content analysis and Bibliometrics analysis
Source: Front Med (Lausanne). 2023 May 5;10:1180550. doi: 10.3389/fmed.2023.1180550 (PMC10196157; doi:10.3389/fmed.2023.1180550)
Supplement: Supplementary file 1 [file Table_1.docx]

**Supplementary Table. Overall function and hierarchical relationships of policy-issuing department**

| **Policymakers** | **Hierarchical relationships** | **Overall function** |
| --- | --- | --- |
| National Health Commission | A State Council department at the ministerial level. | It implements the policies and decisions on health work, formulates national health policies, drafts laws, regulations, policies, and plans for the development of health care. |
| State Council | China's highest national administrative body, with a premier responsibility system. | It leads and coordinates the work of national and local state administrative agencies, formulates administrative measures, regulations, decisions, and orders based on the constitution and laws. |
| National Medical Products Administration | A national bureau managed by the State Administration for Market Regulation (SAMR), at the deputy ministerial level. | It is responsible for guiding the work of provincial, autonomous region, and municipality drug regulatory departments. |
| Ministry of Science and Technology | A State Council department at the ministerial level. | Its responsibilities are to implement the policies and decisions on science and technology innovation and to manage the National Natural Science Foundation Committee (NSFC). |
| Ministry of Human Resources and Social Security | A State Council department at the ministerial level. | Its main function is to implement the policies and decisions on human resources and social security work. |
| National Healthcare Security Administration | A State Council directly affiliated agency at the deputy ministerial level. | Its main function is to implement the policies and decisions on medical security work, and to draft legal regulations, policies, and plans for medical security systems. |
| National Administration of Traditional Chinese Medicine | A national bureau managed by the National Health Commission (NHC), at the deputy ministerial level. | It is the national agency responsible for managing the traditional Chinese medicine industry, formulating strategies, plans, policies, and relevant standards for the development of traditional Chinese medicine and ethnic medicine. |
| Central Committee of the Communist Party of China | The core power structure of the Communist Party of China and the highest leading body of the Party. | It carries out the resolutions of the National People's Congress, leads the work of the Communist Party, and represents the Communist Party of China externally. |
| State Administration for Market Regulation | A State Council directly affiliated agency at the ministerial level. | It is responsible for supervising and managing market order and the National Medical Products Administration (NMPA). |
| Ministry of Civil Affairs | A State Council department at the ministerial level. | It is a department under the State Council responsible for social administrative affairs, and formulates legal regulations, policies, and plans for the development of civil affairs. |
| Ministry of Industry and Information Technology | A State Council department at the ministerial level. | It formulates and organizes the implementation of industry plans and policies in industries such as biomedicine, new materials, aerospace, and information technology. |
| Standing Committee of the National People's Congress | A permanent organ of the National People's Congress, the highest state power organ of the China, exercising state legislative power. | It submits work reports to the National People's Congress, and based on the nomination of the Premier of the State Council, can decide on the appointment and removal of other members of the State Council. |
| China Banking and Insurance Regulatory Commission | A State Council directly affiliated institution at the ministerial level. | Its main responsibility is to supervise and manage the banking and insurance industries in accordance with laws and regulations and to maintain their legal and stable operation. |
| Ministry of Finance | A State Council department at the ministerial level. | Its main functions include formulating financial and taxation development strategies, plans, policies, and reform plans and organizing their implementation. |
| Logistical Support Department of Central Military Commission | The highest command organ responsible for military logistics professional services under the leadership of the Central Military Commission (CMC). | Its main responsibility is to perform logistical planning, policy research, standard formulation, inspection, and supervision for the entire army. |
| China Disabled Persons' Federation | A national mass organization directly affiliated with the State Council at the ministerial level. | Its main responsibility is to coordinate the formulation and implementation of the State Council's policies, regulations, plans, and planning related to disability work. |
| National Development and Reform Commission | A State Council department at the ministerial level. | Its main responsibility is to implement the policies and decisions of the Party Central Committee on development and reform work, formulate and organize the implementation of national economic and social development strategies. |
| General Administration of Sport | A State Council directly affiliated agency at the ministerial level. | Its main responsibility is to formulate sports development plans and policies, draft relevant legal regulations, and supervise their implementation. |
| General Administration of Customs | A State Council directly affiliated agency at the ministerial level. | Its main responsibility is to implement the policies and decisions of the Party Central Committee on customs work, be responsible for health quarantine for entry and exit, and the entry and exit of animals and plants. |
| Ministry of Education | A State Council department at the ministerial level. | Its main responsibility is to research and formulate guidelines and policies for education work, as well as draft laws and regulations related to education. |
| State Taxation Administration | A State Council directly affiliated institution at the ministerial level. | Its primary responsibility is to draft tax laws, regulations, and implementation rules, as well as propose tax policy recommendations. |
| Chinese Academy of Sciences | A State Council directly affiliated institution at the ministerial level. | It is the highest academic institution for natural sciences, the top advisory body for scientific and technological matters, and a comprehensive research and development center for natural sciences and advanced technologies in China. |
| Chinese Academy of Engineering | A State Council directly affiliated institution at the ministerial level. | It is the highest honorary and advisory academic institution in China's engineering science and technology field, implementing policies and decisions regarding the construction of a team of academicians in the engineering and technology field and strategic consultation. |
| National Natural Science Foundation | A deputy ministerial-level institution managed by the Ministry of Science and Technology（MOST）. | Its main responsibility includes providing support for fundamental scientific research, nurturing and cultivating scientific and technological expertise, advancing scientific and technological advancement, and continually enhancing the nation's ability to independently innovate. |
